# Fyn amplifies NLRP3 inflammasome signaling in Parkinson's disease

**DOI:** 10.18632/aging.102210

**Published:** 2019-08-27

**Authors:** Nikhil Panicker, Arthi Kanthasamy, Anumantha G. Kanthasamy

**Affiliations:** 1 Neuroregeneration and Stem Cell Programs, Institute for Cell Engineering, Johns Hopkins University School of Medicine, Baltimore, MD 21205, USA; 2 Department of Neurology, Johns Hopkins University School of Medicine, Baltimore, MD 21205, USA; 3 Parkinson Disorders Research Program, Iowa Center for Advanced Neurotoxicology, Department of Biomedical Sciences, Iowa State University, Ames, IA 50011, USA

**Keywords:** Fyn, brain inflammation, inflammasome, neurodegenerative diseases, aging

Parkinson’s disease (PD) is an aging-associated, progressive and neurodegenerative disorder characterized by the slow demise of dopamine-producing neurons in the substantia nigra (SN). Intracytoplasmic inclusions rich in misfolded α-synuclein (αSyn), resulting from age-impaired protein degradation machinery, are the major histopathological characteristic of PD. Various lines of evidence from mouse models, post-mortem analysis of PD brains, and genome-wide association studies (GWAS) implicate chronic, microglia-mediated sterile neuroinflammation as a crucial contributing factor in the progression of PD [1–3]. Misfolded/aggregated αSyn instigates neuroinflammatory responses of microglia by acting as a strong endogenic antigen [4,5]. The nod-like receptor protein-3 (NLRP3) inflammasome is a multi-protein complex that constitutes a major arm of the innate immune system. Like other inflammasomes, activation of the NLRP3 inflammasome culminates in the autoproteolytic cleavage of caspase-1 (Casp1), which in turn cleaves the pro-cytokine pro-interleukin-1 beta (pro-IL-1β) to mature IL-1β. However, the NLRP3 inflammasome is unique in its evolution, in that it requires two steps for its activation: (1) priming, which is mediated by nuclear factor kappa B (NFκB) pathway activation and involves the induction of pro-IL-1β and NLRP3 proteins, and (2) activation, which entails the uptake of a pathogen or damage-associated signal. This second step results in the generation of mitochondria-derived reactive oxygen species (mitoROS), leading to inflammasome assembly/activation. Hyperactivation of the NLRP3 inflammasome has been demonstrated in several neurodegenerative diseases, including PD [5]. In most articles that study NLRP3 signaling, the fulfillment of the priming and activation steps is accomplished by two distinct agents. Bacteria-derived lipopolysaccharide (LPS) is usually used as the priming signal. However, the validity of using a microbial component such as LPS to model the sterile neuroinflammation that characterizes neurodegenerative diseases has been called into question. In our recent article, we uncovered that aggregated αSyn can simultaneously prime and activate the NLRP3 inflammasome in microglia [6]. This dual action of αSyn portends its neuroinflammatory properties through the sustained activation of NLRP3 inflammasome signaling to drive the chronic state of PD progression.

The proximal signaling mechanisms that govern microglial inflammasome assembly in neurodegenerative diseases are largely uncharacterized. We [7] previously demonstrated the role of the non-receptor Src family kinase Fyn in regulating microglial activation in PD models, wherein inflammogen stimulation of microglia rapidly elicits Fyn activation. The activated Fyn gets tyrosine-phosphorylated and activates the pro-inflammatory protein kinase C-delta (PKCδ), contributing to p65-NFκB nuclear translocation and pro-inflammatory gene transcription. In our recent study [6], we further discovered that the Fyn-PKCδ axis also contributes to NFκB activation upon αSyn stimulation of microglia, leading to inflammasome priming and the induction of pro-IL-1β and NLRP3 mRNA and protein levels. More interestingly, we show that Fyn augments the second step of NLRP3 inflammasome assembly by associating with the scavenger receptor CD36 and mediating αSyn uptake into microglia. Moreover, the microglial αSyn uptake occurs independent of PKCδ. Thus, Fyn is a major regulator of αSyn-mediated inflammasome priming as well as activation. Next, we demonstrated activation of the NLRP3 inflammasome in the adeno-associated viral (AAV) αSyn and the A53T-αSyn mouse models of PD, as well as in human PD brain lysates. AAV αSyn-injected Fyn-deficient mice displayed strongly attenuated neuroinflammation and microglial NLRP3 inflammasome activation when compared to wild-type (WT) control mice. Since the NLRP3 inflammasome is hyperactivated in PD, and Fyn governs microglial inflammasome assembly, we examined whether Fyn was activated under PD conditions. Immunoblot and immunohistochemical studies revealed induction of both native and activated Fyn within the ventral midbrain microglia of PD brains (when compared to age-matched control brains), further confirming Fyn’s pathological role in PD. Remarkably, as this article was being written for publication, the FYN gene was independently found to be associated with PD in a GWAS [3].

**Figure 1:**
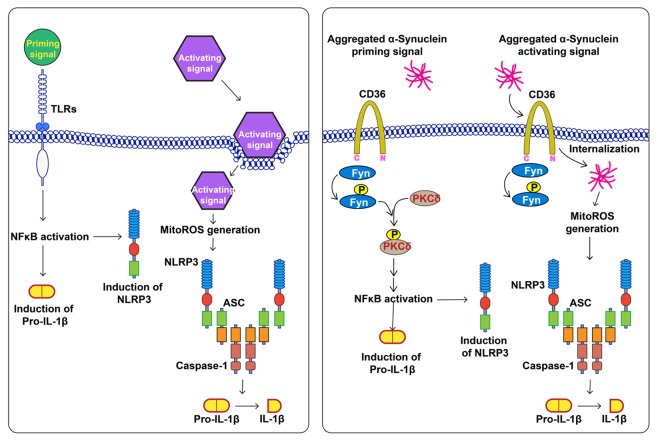
**Role of Fyn in misfolded α-synuclein (αSyn)-mediated NLRP3 inflammasome activation**. Left panel: The typical activation mechanism of the NLRP3 inflammasome, wherein disparate priming and activating signals are required for NLRP3 inflammasome assembly. The priming signal activates the NFκB pathway, leading to the elevated expression of NLRP3 and pro-IL-1β. The activation signal causes the release of mitoROS, which triggers inflammasome assembly, leading to cleavage of pro-IL-1β to IL-1β, which gets secreted. Right panel: αSyn can prime the NLRP3 inflammasome *via* CD36-Fyn-PKCδ signaling, which is required for NFκB activation, and can activate the NLRP3 inflammasome upon its uptake mediated by CD36 and Fyn.

After having established the role of Fyn in PD-associated NLRP3 inflammasome priming and activation, we will next explore the efficacy of Fyn inhibitors to inhibit neuroinflammation and consequent neurodegeneration in αSyn PD models (Figure 1). Moreover, it remains to be demonstrated how inflammasome activation in PD contributes to the death of SN dopaminergic neurons. This could potentially be effectuated by the inflammasome-associated cytokine IL-1β, which has pleiotropic roles including amplifying pro-inflammatory cascades and directly causing neuron death [8], or by hitherto undiscovered mechanisms. In summary, we suggest that Fyn kinase has emerged as a major driver of the neuroinflammatory process in PD, and one that can be further exploited as a translational therapeutic target to develop novel neuroprotective agents for PD as well as other aging-associated proteinopathies including Alzheimer’s disease.
